# Association of high kinesiophobia and pain catastrophizing with quality of life in severe hip osteoarthritis: a cross-sectional study

**DOI:** 10.1186/s12891-023-06496-6

**Published:** 2023-05-16

**Authors:** Ryo Hidaka, Takeyuki Tanaka, Kazuaki Hashikura, Hiroyuki Oka, Ko Matsudaira, Toru Moro, Kenta Matsuda, Hirotaka Kawano, Sakae Tanaka

**Affiliations:** 1 Department of Orthopaedic Surgery, Teikyo University School of Medicine, 2-11-1 Kaga, Itahashi-ku, Tokyo, 173-8606 Japan; 2 Division of Science for Joint Reconstruction, Graduate School of Medicine, The University of Tokyo, 7-3-1 Hongo, Bunkyo-ku, Tokyo, 113-8655 Japan; 3 Department of Medical Research and Management for Musculoskeletal Pain, 22nd Century Medical and Research Center, Faculty of Medicine, The University of Tokyo, 7-3-1 Hongo, Bunkyo-ku, Tokyo, 113-8655 Japan; 4 Department of Orthopaedic Surgery, Faculty of Medicine, , The University of Tokyo, 7-3-1 Hongo, Bunkyo-ku, Tokyo, 113-8655 Japan

**Keywords:** Osteoarthritis, Kinesiophobia, Pain catastrophizing, Quality of life, Tampa scale for kinesiophobia, Pain catastrophizing scale

## Abstract

**Background:**

While fear of movement is an important predictor of pain and disability in osteoarthritis (OA), its impact on patients with hip OA remains uncertain. This study aimed to determine whether fear of movement, evaluated by the Tampa Scale for Kinesiophobia (TSK)-11, and pain catastrophizing, evaluated by the Pain Catastrophizing Scale (PCS), were associated with quality of life (QOL) in patients with hip OA.

**Methods:**

This cross-sectional study was conducted between November 2017 and December 2018. Ninety-one consecutively enrolled patients with severe hip OA were scheduled for primary unilateral total hip arthroplasty. The EuroQOL-5 Dimensions questionnaire was used to measure general QOL. The Japanese Orthopedic Association Hip Disease Evaluation Questionnaire was used to assess disease-specific QOL. The covariates included age, sex, body mass index (BMI), pain intensity, high pain catastrophizing (PCS ≥ 30), and high kinesiophobia (TSK-11 ≥ 25). Variables were subjected to multivariate analysis using each QOL scale.

**Results:**

In multiple regression analysis, pain intensity, high pain catastrophizing, and BMI were independently correlated with the disease-specific QOL scale. High pain catastrophizing, pain intensity, and high kinesiophobia were independently correlated with the general QOL scale.

**Conclusions:**

High pain catastrophizing (PCS ≥ 30) was independently associated with disease and general QOL scales. High kinesiophobia (TSK-11 ≥ 25) was independently associated with the general QOL scale in preoperative patients with severe hip OA.

## Background

Osteoarthritis (OA) is a common chronic disease that causes pain, functional limitations, and a reduced quality of life (QOL) among adults worldwide [1, 2]. The fear-avoidance model was proposed to explain how pain, physical disability, and affective distress develop as a result of persistent fear-motivated avoidance behavior in chronic musculoskeletal pain [3]. This model proposed that pain perception was primarily influenced by pain catastrophizing and pain-related fear of movement [4]. The importance of these two cognitive parameters in predicting pain and disability in OA has been supported in other studies.

Recent studies reported that pain catastrophizing in knee OA is associated with daily physical activity [5, 6], walking speed [7], and pain intensity [8, 9], and it can significantly reduce disease-specific QOL [9]. Similarly, fear of movement in knee OA was reportedly associated with physical activity [6], self-reported physical function [10], psychological disability, slower gait speed [11], and disease-specific QOL [12]. Thus, most of the previous studies have investigated associations between pain catastrophizing and QOL in knee OA, a condition more common in older adults; and research on the influence of psychological factors in patients with hip pathology is limited. A lower physical function and disease-specific QOL have been reported in patients with hip OA compared to those with knee OA [13, 14]; therefore patients with knee OA and hip OA should be considered separately. In a study conducted on patients with hip OA, it was found that pain catastrophizing was independently associated with disease-specific and general QOL in preoperative patients with severe hip OA [15]. Higher reported subjective function in activities of daily living (ADL) was associated with lower pain catastrophizing in hip pathology [16]. Therefore, though it is known that pain catastrophizing is associated with QOL and ADL in patients with hip OA, the role of fear of movement remains unclear. In a previous study on the association between pain catastrophizing and QOL in patients with hip OA [15], multivariate analyses have included only pain catastrophizing but not the fear of movement as a cognitive parameter. The independent association of pain catastrophizing and kinesiophobia with QOL remains unclear; clarification of this association may improve the QOL by improving these cognitive parameters through appropriate psychological interventions [17, 18].

This study aimed to investigate the effect of fear of movement with the Tampa Scale for Kinesiophobia (TSK)-11 and pain catastrophizing with the Pain Catastrophizing Scale (PCS) on both general and disease-specific QOL scales in patients with severe hip OA.

## Methods

### Participants

This cross-sectional study was approved by the institutional review board of the University of Tokyo Hospital, with the ethical approval number: 11,725-(1), and was conducted in accordance with the World Medical Association Declaration of Helsinki. We included 105 patients with hip OA, who were scheduled to undergo primary unilateral total hip arthroplasty (THA) at our institution from November 2017 to December 2018. Written informed consent for all procedures was obtained from all patients at hospital admission. The inclusion criteria were: (1) diagnosis of hip OA by an orthopedic surgeon through clinical examinations and radiographic findings using the American College of Rheumatology criteria; (2) radiographic hip OA > grade 3 using radiographic images on the Kellgren–Lawrence criteria; and (3) presence of hip pain for at least six months and functional limitations in ADL that required THA. We excluded 14 patients for the following reasons: inability to obtain informed consent (n = 3); incomplete response to the questionnaires (n = 5); complications of psychiatric disorders such as schizophrenia (n = 4); and presence of severe knee OA compatible with hip pain severity (n = 2). Finally, 91 patients with severe hip OA were evaluated in this study.

### Measures

#### Patient demographics

We investigated patients’ demographic data, including age, sex, body mass index (BMI), and smoking history. Validated questionnaires were completed by each participant after admission, before surgery.

#### Pain intensity

Pain intensity was measured using a visual analog scale (VAS), which is a component of the Japanese Orthopaedic Association Hip Disease Evaluation Questionnaire (JHEQ) [19]. VAS, a 100-mm line anchored by two verbal descriptors (i.e., for instance “no pain” and “worst imaginable pain”), is widely used for estimating pain severity as well as judging the extent of pain relief in clinical pain research [20, 21]. This scale has been reported as reliable and valid for measuring pain intensity [22].

#### Pain catastrophizing

Pain catastrophizing was assessed with the 13-item PCS, a validated and widely used instrument for measuring pain-related catastrophic thinking [23, 24]. Participants responded to each item using a 5-point Likert scale (0 = “not at all”, 4 = “all the time”). This scale provides a total score and three subscales: rumination (four items), magnification (three items), and helplessness (six items). Total PCS score ranges from 0 to 52, with a higher score indicating greater pain catastrophizing. As per the PCS user manual, a total PCS score of 30 is clinically significant. The Japanese version of this scale has been reported as reliable and valid [25].

#### Tampa scale for kinesiophobia (TSK)

Fear of movement was assessed using the Japanese version of the previously validated TSK-11 [26]. TSK-11 comprises 11 items, each of which is rated on a 4-point Likert scale (1 = strongly disagree, 4 = strongly agree). The scores were summed (range: 11 to 44), with a higher total score indicating a greater degree of pain-related fear of movement [27]. A TSK-11 score ≥ 25 was considered indicative of excessive kinesiophobia [28].

#### Quality of life (QOL)

QOL was assessed with two patient-record outcome measures. The EuroQOL-5 Dimensions (EQ-5D)-3 L is a generic health-related QOL instrument tool with available local Japanese set value [29]. This questionnaire describes the respondent’s health state with three severity levels (no problems, some problems, or serious health problems) in each of the five dimensions: mobility, usual activities, self-care, pain/discomfort, and anxiety/depression. Two hundred forty-three health statuses could be determined by calculating the EQ-5D scores (range: -0.111 to 1.000). Negative scores represent a health state worse than being dead, 0 represents dead, and 1.000 represents a state of full health.

The JHEQ was used to evaluate disease-specific QOL in patients with hip joint disease. This questionnaire included questions related to movements specific to the Asian lifestyle, such as the use of a Japanese-style toilet and getting up from the floor. As such, JHEQ is a useful tool for evaluating Japanese patients with hip disease. This questionnaire has also been reported as reliable and valid [30]. JHEQ consists of pain, movement, and mental subscales. The score for each subscale ranges from 0 to 28 points, with a maximum of 84 points. Higher scores indicate better results. The patients’ hip pain was measured using VAS, ranging from 0 mm (completely satisfied or no pain at all) to 100 mm (completely dissatisfied or maximum pain). VAS score for hip pain was converted to a 0-to-4-point scale (0 = 81–100 mm, 1 = 61–80 mm, 2 = 41–60 mm, 3 = 21–-40 mm, and 4 = 0–20 mm), with each question on each subscale having a score of 0–4 points (0 = strongly agree, 1 = agree, 2 = uncertain, 3 = disagree, and 4 = strongly disagree). While the scores were calculated separately for the right and left sides, the score for the side with the hip problem was used.

### Sample size

The sample size was estimated using G*Power 3.1.9.6 for Mac (G*Power© from the University of Dusseldorf, Germany) [31]. More specifically, the sample size was calculated using multiple linear regression (for instance, fixed model with R2 deviation from zero). A medium effect size of 0.20 was used to obtain 80% statistical power (1-β error probability) with an α error level probability of 0.05. Six predictors were selected. Ultimately, we estimated that a minimum of 75 participants would be required for this study.

### Statistical analysis

Demographic variables were presented as medians and interquartile ranges (IQR). Patients were stratified into groups based on a predefined cut-off scores for PCS and TSK-11. Fisher’s exact test was used to compare categorical parameters, and the Wilcoxon rank-sum test was used to compare continuous parameters between the groups. Correlation of QOL scores (EQ-5D and JHEQ) with each variable was analyzed using the Spearman’s rank correlation coefficient test. Factors affecting QOL scores were determined. Each QOL score (EQ-5D and JHEQ) was set as a dependent variable. VAS score; high pain catastrophizing (PCS ≥ 30), of which association with QOL was previously reported [15]; high kinesiophobia (TSK-11 ≥ 25); and potential confounding factors, including age, sex, and BMI, were set as independent variables. Statistical significance threshold was defined as *P* < 0.05. Statistical analyses were performed using the JMP software (version 14.0; SAS Institute, Cary, NC, USA).

## Results

**Fig. 1:**
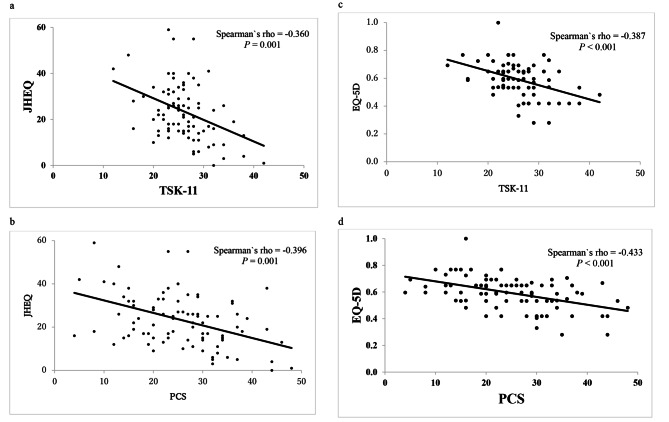
Scatter plot diagram showing the correlation of QOL with TSK-11 and PCS. **(a)** Scatter plots show the negative correlation between JHEQ and TSK-11 results. **(b)** Scatter plots show the negative correlation between JHEQ and PCS results. **(c)** Scatter plots show the negative correlation between EQ-5D and TSK-11 results. **(D)** Scatter plots show the negative correlation between EQ-5D and PCS results. QOL, quality of life; EQ-5D, EuroQOL-5 Dimensions; JHEQ, Japanese Orthopaedic Association Hip Disease Evaluation Questionnaire; PCS, pain catastrophizing scale; TSK, Tampa Scale for Kinesiophobia

Patient demographics and outcomes are summarized in Table 1. Data of 91 patients (76 women and 15 men, median age: 65 years) were reviewed. The median TSK-11 and PCS scores were 26 and 25, respectively. Fifty-six patients (62%) had high kinesiophobia (TSK-11 ≥ 25). Thirty-one patients (34%) experienced high pain catastrophizing (PCS ≥ 30). The results of patients stratified by the level (low and high) of kinesiophobia and pain catastrophizing are shown in Table 2. The high kinesiophobia group had significantly lower JHEQ total, JHEQ movement, JHEQ mental, and EQ-5D scores than the low kinesiophobia group (*P* = 0.003, *P* = 0.035, *P* = 0.001, and *P* = 0.009, respectively). There was no significant difference in VAS score between two groups. Patients with high pain catastrophizing had significantly lower JHEQ total, JHEQ pain, JHEQ movement, JHEQ mental, and EQ-5D scores than those with low pain catastrophizing (*P* < 0.001, *P* < 0.001, *P* = 0.009, *P* < 0.001, and *P* < 0.001, respectively). VAS and TSK-11 scores in patients with high pain catastrophizing were significantly higher than those with low pain catastrophizing (*P* < 0.005 for both). Correlations between each QOL scale showed a significant difference in VAS, TSK-11, and PCS scores (all *P* ≤ 0.001) (Table 3; Fig. 1).

**Table 1:** Demographic characteristics and outcomes of the study participants

Variables	Median (IQRs)
**Age, years**	65 (58–74)
**Female sex, n (%)**	76 (84)
**BMI, kg/m** ^**2**^	23.4 (21.1–26.5)
**Smoking history, n (%)**	16 (18)
**VAS**	79 (64–88)
**JHEQ total**	22 (15–32)
**Pain**	8 (4–12)
**Movement**	4 (1–8)
**Mental**	9 (5–13)
**EQ-5D**	0.596 (0.533–0.649)
**TSK-11**	26 (23–29)
**PCS total**	25 (19–32)
**Rumination**	14 (10–16)
**Magnification**	4 (3–6)
**Helplessness**	7 (5–11)

Data are presented as medians (IQR). BMI, body mass index; EQ-5D, EuroQOL-5 Dimensions; IQR, interquartile range; JHEQ, Japanese Orthopaedic Association Hip Disease Evaluation Questionnaire; PCS, pain catastrophizing scale; TSK, Tampa Scale for Kinesiophobia; VAS, visual analog scale

**Table 2:** Differences in demographic and outcome characteristics of patients stratified by the level (high and low) of kinesiophobia and pain catastrophizing

	TSK-11 < 25 n = 35	TSK-11 ≥ 25 n = 56	*P*-value		PCS < 30 n = 60	PCS ≥ 30 n = 31	*P*-value
**Age, years**	65 (56–70)	66 (58–75)	0.37		65 (58–71)	66 (58–76)	0.52
**Female sex, n (%)**	33 (94)	43 (77)	0.04		51 (85)	25 (81)	0.77
**BMI, kg/m** ^**2**^	21.9 (19.9–24.9)	24.0 (21.4–26.9)	0.05		23.2 (20.9–26.3)	24.1 (21.1–26.5)	0.46
**VAS**	79 (62–86)	79.5 (65–88.8)	0.48		72.5 (60.8–85)	86 (79–93)	0.003
**Smoking history, n (%)**	6 (17)	10 (18)	0.93		12 (20)	4 (13)	0.56
**JHEQ total**	27 (18–34)	17.5 (12.3–26)	0.003		26 (17.3–34.8)	15 (6–22)	< 0.001
**Pain**	10 (5–12)	6 (3.3–12)	0.14		10 (6–12)	4 (2–7)	< 0.001
**Movement**	5 (2–9)	3 (1–7)	0.035		5 (2–9)	2 (0–5)	0.009
**Mental**	13 (9–16)	8 (5–11.8)	0.001		11.5 (8–15)	6 (3–9)	< 0.001
**EQ-5D**	0.649 (0.587–0.705)	0.587 (0.419–0.649)	0.009		0.649 (0.587–0.693)	0.533 (0.419–0.596)	< 0.001
**TSK-11**					24 (23–27)	28 (26–32)	< 0.001
**PCS**	21 (15–25)	29 (22–33.8)	< 0.001				

Data are presented as medians (IQR). BMI, body mass index; EQ-5D, EuroQOL-5 Dimensions; IQR, interquartile range; JHEQ, Japanese Orthopaedic Association Hip Disease Evaluation Questionnaire; PCS, pain catastrophizing scale; TSK, Tampa Scale for Kinesiophobia; VAS, visual analog scale

**Table 3:** Univariate analysis between continuous variables and QOL scores (EQ-5D and JHEQ)

Variables	JHEQ		EQ-5D	
	Rho	*P-*value	Rho	*P-*value
**Age**	-0.196	0.067	− 0.0711	0.51
**BMI**	-0.172	0.11	− 0.0329	0.76
**VAS**	-0.522	< 0.001	− 0.456	< 0.001
**TSK-11**	-0.360	0.001	− 0.387	< 0.001
**PCS total**	-0.396	0.001	− 0.433	< 0.001
**Rumination**	− 0.381	< 0.001	− 0.402	< 0.001
**Magnification**	− 0.293	0.0048	− 0.313	0.003
**Helplessness**	− 0.434	< 0.001	− 0.457	< 0.001

BMI, body mass index; QOL, quality of life; EQ-5D, EuroQOL-5 Dimensions; JHEQ, Japanese Orthopaedic Association Hip Disease Evaluation Questionnaire; PCS, pain catastrophizing scale; TSK, Tampa Scale for Kinesiophobia; VAS, visual analog scale

In multivariate analysis, VAS score, high pain catastrophizing, and BMI were significantly associated with JHEQ score (*P* < 0.001, *P* = 0.002, and *P* = 0.023, respectively) (Table 4). EQ-5D score was significantly associated with VAS score, high pain catastrophizing, and high kinesiophobia (*P* = 0.01, *P* < 0.001, *P* = 0.024, respectively) (Table 5). In the multiple regression model of JHEQ, VAS standardized beta value was the highest (-0.46), followed by those of high pain catastrophizing (-0.28) and BMI (-0.18). In the multiple regression model of EQ-5D, the standardized beta value for high pain catastrophizing was the highest (-0.36), followed by those for VAS (-0.24) and high kinesiophobia (-0.23).

**Table 4:** Multiple regression analysis for JHEQ

Variables	B	SE	Beta	*P-*value	R^2^
					0.475
**Age**	-0.170	0.099	-0.137	0.088	
**Female sex**	0.701	1.372	0.041	0.61	
**BMI**	-0.598	0.258	-0.184	0.023	
**VAS**	-0.243	0.043	-0.459	< 0.001	
**TSK-11** ≥ **25**	-1.361	1.14	-0.104	0.23	
**PCS** ≥ **30**	-3.703	1.14	-0.276	0.002	

JHEQ, Japanese Orthopaedic Association Hip Disease Evaluation Questionnaire; B, nonstandard regression coefficient; Beta, standardized regression coefficient; BMI, body mass index; PCS, pain catastrophizing scale; R^2^, multiple correlation coefficient adjusted for degrees of freedom; SE, standard error; TSK, Tampa Scale for Kinesiophobia; VAS, visual analog scale

**Table 5:** Multiple regression analysis for EQ-5D

Variables	B	SE	Beta	*P-*value	R^2^
					0.315
**Age**	0.0004	0.001	0.036	0.68	
**Female sex**	0.015	0.015	0.091	0.32	
**BMI**	0.0014	0.0028	0.044	0.63	
**VAS**	-0.0012	0.00046	-0.24	0.01	
**TSK-11** ≥ **25**	-0.028	0.012	-0.23	0.024	
**PCS** ≥ **30**	-0.046	0.012	-0.36	< 0.001	

EQ-5D, EuroQOL-5 Dimensions; B, nonstandard regression coefficient; Beta, standardized regression coefficient; BMI, body mass index; PCS, pain catastrophizing scale; R^2^, multiple correlation coefficient adjusted for degrees of freedom; SE, standard error; TSK, Tampa Scale for Kinesiophobia; VAS, visual analog scale

## Discussion

In this study, high pain catastrophizing (PCS ≥ 30) was associated with both disease-specific and general QOL in patients with severe hip OA before THA. High kinesiophobia (TSK-11 score ≥ 25) was associated with the general QOL scale. High pain catastrophizing had the worst or second-worst QOL, followed by pain intensity, depending on the scale used. High kinesiophobia had the third-worst general QOL after pain intensity and pain catastrophizing. High pain catastrophizing and high kinesiophobia were associated factors for QOL in patients with severe hip OA.

A cross-sectional study on severe hip OA reported that pain catastrophizing was independently associated with disease-specific and general QOL scales [15]. However, this study neither assessed kinesiophobia, nor analyzed PCS score as a continuous variable, and did not stratify patients by cut-off values. The PCS user manual defines that a total PCS score of 30 represents clinically relevant level of catastrophizing, and that this score corresponds to the 75th percentile of the distribution of PCS scores in chronic pain patients; this cut-off score was also used by another study on hip pathology [16]. Therefore, a PCS cut-off value of 30 has been used and analyzed in the present study. Our study included the assessment of high kinesiophobia and suggested that high pain catastrophizing (PCS ≥ 30) was strongly associated with disease-specific and general QOL scales. Our median PCS scores (25 points) was comparable with that of the previous study (26 points) [15], suggesting that the psychological background of patients with severe hip OA scheduled to undergo THA was similar in these two studies and therefore, did not affect the results. However, the number of patients in each study was small, and comprised only of Japanese participants. A systematic review in patients with chronic primary pain have reported PCS scores being significantly higher in Asian populations compared to Western populations [32]. A meta-analysis of the association of PCS with participant characteristics have revealed that the lower limb pain tended to show low PCS compared to other regions [33]. In this study, a PCS cutoff value of 30 was used based on the PCS user manual in chronic pain patients, however it is still not clear whether this cut-off value is suitable for hip OA in the Japanese population, and future studies of a PCS cut-off value in patients with hip OA in countries situated in various geographical regions are needed. We postulate that QOL is affected in patients with high pain catastrophizing and a PCS score ≥ 30.

Two studies evaluated kinesiophobia in patients who underwent THA. One study showed that patients with high kinesiophobia (TSK-13 ≥ 40) had higher preoperative Western Ontario and McMaster Universities Osteoarthritis Index (WOMAC) total and functional scores [34]. In the preoperative phase, patients with high kinesiophobia exhibited more impaired preoperative functional abilities. The proportion of patients with preoperative high kinesiophobia was 30%. Another study reported a proportion of preoperative high kinesiophobia (defined as TSK-17 ≥ 40) of approximately 50% [35]. In this study, high kinesiophobia was defined as TSK-11 ≥ 25, and the proportion of high kinesiophobia was 62%, which is higher than those from other reports. If the TSK-11 cut-off values were set higher, the results could have been different. In the 17-item TSK, the total score ranges from 17 to 68, with a score > 37 generally indicating a high level of kinesiophobia [36]. If a score of 37 (57%) on the 68-graded scale represents high kinesiophobia, it would be equal to 35 points on the 44-point TSK-11 scale [37]. Since our study had very few patients with TSK-11 ≥ 35 (4/91), this cut-off value was unsuitable for evaluation. To the best of our knowledge, only one study on TSK-11 cut-off scores (TSK-11 ≥ 25) in patients with knee pathology is available [28]. The validity of this cut-off scores for patients with hip OA remains unclear; therefore, further research are needed to investigate cut-off scores for both PCS and TSK-11 in this population. A few reports on TSK-11 have shown a mean score of 24.5 for patients with fibromyalgia and OA, 27.7 for those with chronic low back pain [38], and 26.22 for preoperative patients who underwent hip arthroscopy in femoroacetabular impingement [39]. These values are consistent with the median score of 26 in our study.

Pain intensity was not significantly different between the high and low kinesiophobia groups in this study. Several studies reported an association between pain intensity and kinesiophobia. The intensity of activity-related and resting pain was associated with the TSK-17 score in patients with knee OA [40]. However, two reports showed no correlation between pain intensity and kinesiophobia in knee OA [12, 41]. In patients before THA, the WOMAC pain score was not significantly different between the high and low kinesiophobia groups [34]. Our study of hip OA indicated that reducing pain alone may not be sufficient to reduce kinesiophobia, which may be an important finding. Since pain intensity was strongly associated with each QOL scale, kinesiophobia unrelated to pain intensity may have had a weaker association with QOL.

BMI was associated with disease-specific QOL scores, followed by pain intensity and high pain catastrophizing in our study, suggesting that BMI is a factor that can impair QOL. Several studies reported an association between BMI and hip OA. In this regard, a retrospective cohort study reported that BMI had an independent, weak negative impact on health-related QOL in patients with hip OA [42]. BMI was the only factor that could be intervened in the preoperative phase for an improvement of early functional performance after THA [34]. A systematic review showed that a low level of physical function was associated with a higher BMI in hip OA [43]. In other systematic reviews of THA, preoperative BMI was a significant factor in some reports [44, 45], but not others [46]. Therefore, the role of BMI in hip OA remains unclear.

High kinesiophobia and high pain catastrophizing were independent associated factors of QOL in this study. We suggested that efforts to increase QOL in patients with severe hip OA before THA may be enhanced by strategies aiming to reduce the fear of movement and pain catastrophizing. However, this was a cross-sectional study, and longitudinal studies are required to investigate the association of kinesiophobia, pain catastrophizing, and other psychological factors with QOL. There is evidence that specific pain neurophysiology education could reduce pain catastrophizing and increase knowledge about pain in people with chronic pain [47]. There are a few studies on cognitive behavioral therapy [18, 48], education, and graded exposure [49] for the treatment of fear of movement in patients with chronic pain. Such psychological interventions may improve QOL in patients with severe hip OA before THA. There are also two studies of the association between outcome after THA and kinesiophobia [34, 35]. However, there are no studies of the association between outcome after THA and pain catastrophizing. Early functional performance after THA was not correlated with kinesiophobia level [34]. Providing individual support and attention to patients undergoing the surgical procedure and the rehabilitation of patients after THA, who have low self-efficacy, high fear of motion, or both, reduce their hospital length of stay [35]. Whether the two psychological factors of pain catastrophizing and fear of movement impact outcomes after THA remain uncertain. Since our study did not include postoperative assessment after THA, further studies are needed.

This study had several limitations. First, it included only a small number of patients from a single center; therefore, its generalizability remains unclear. Second, while we only analyzed pain catastrophizing and fear of movement, other psychological factors (depression, anxiety, patient expectations, and self-efficacy) have been evaluated in a systematic review of the outcomes of THA and total knee arthroplasty [50]. Third, the JHEQ in this study was not created for use in regions of western culture. Fourth, an earlier study had revealed that educational attainment was associated with health-related QOL [51], which was not examined in this study.

## Conclusion

High kinesiophobia (TSK-11 ≥ 25) was associated only with general QOL in patients with preoperative severe hip OA. High pain catastrophizing (PCS ≥ 30) was associated with both, disease-specific and general QOL. This study suggested that screening for individual psychological factors, such as pain catastrophizing and fear of movement, should be considered, and therapeutic intervention should be assessed to improve QOL in patients with severe hip OA.

## Data Availability

The datasets used and analyzed during the current study are available from the corresponding author on reasonable request.

## Abbreviations

OA: Osteoarthritis
QOL: Quality of life
ADL: Activities of daily living
TSK: Tampa Scale for Kinesiophobia
PCS: Pain Catastrophizing Scale
THA: Total hip arthroplasty
BMI: Body mass index
VAS: Visual analog scale
JHEQ: Japanese Orthopaedic Association Hip Disease Evaluation Questionnaire
EQ-5D: EuroQOL-5 Dimensions
IQR: Interquartile range
WOMAC: Western Ontario and McMaster Universities Osteoarthritis Index
